# Engineering the interactions between a plant‐produced HIV antibody and human Fc receptors

**DOI:** 10.1111/pbi.13207

**Published:** 2019-08-10

**Authors:** Szymon Stelter, Mathew J. Paul, Audrey Y.‐H. Teh, Melanie Grandits, Friedrich Altmann, Jessica Vanier, Muriel Bardor, Alexandra Castilho, Rachel Louise Allen, Julian K‐C. Ma

**Affiliations:** ^1^ Hotung Molecular Immunology Unit Institute for Infection and Immunity St George's University of London London UK; ^2^ Division of Biochemistry University of Natural Resources and Life Sciences Vienna Austria; ^3^ UNIROUEN Laboratoire Glycobiologie et Matrice Extracellulaire Végétale EA Normandie Univ Rouen France; ^4^ Institut Universitaire de France (I.U.F.) Paris Cedex 05 France; ^5^ Department of Applied Genetics and Cell Biology University of Natural Resources and Life Sciences Vienna Austria; ^6^ Institute for Infection and Immunity St George's University of London London UK; ^7^ Present address: Crescendo Biologics Ltd Meditrina Building 260 Babraham Research Campus Cambridge CB22 3AT UK

**Keywords:** antibody, glycoengineering, plant, molecular pharming, fucose, Fc receptor, CD64, CD16, FcRn, neonatal Fc receptor, methionine oxidation

## Abstract

Plants can provide a cost‐effective and scalable technology for production of therapeutic monoclonal antibodies, with the potential for precise engineering of glycosylation. Glycan structures in the antibody Fc region influence binding properties to Fc receptors, which opens opportunities for modulation of antibody effector functions. To test the impact of glycosylation in detail, on binding to human Fc receptors, different glycovariants of VRC01, a broadly neutralizing HIV monoclonal antibody, were generated in *Nicotiana benthamiana* and characterized. These include glycovariants lacking plant characteristic α1,3‐fucose and β1,2‐xylose residues and glycans extended with terminal β1,4‐galactose. Surface plasmon resonance‐based assays were established for kinetic/affinity evaluation of antibody–FcγR interactions, and revealed that antibodies with typical plant glycosylation have a limited capacity to engage FcγRI, FcγRIIa, FcγRIIb and FcγRIIIa; however, the binding characteristics can be restored and even improved with targeted glycoengineering. All plant‐made glycovariants had a slightly reduced affinity to the neonatal Fc receptor (FcRn) compared with HEK cell‐derived antibody. However, this was independent of plant glycosylation, but related to the oxidation status of two methionine residues in the Fc region. This points towards a need for process optimization to control oxidation levels and improve the quality of plant‐produced antibodies.

## Introduction

Antibody‐based therapeutics comprise a major segment of the fast‐growing biopharmaceutical market. There are currently more than 50 monoclonal antibodies approved for clinical use, and hundreds are in preclinical and clinical development (Ecker *et al*., 2015). Most of these are IgG antibodies that target cancer and autoimmune disorders, although there is growing interest in monoclonal antibodies to tackle infectious diseases (Irani *et al*., 2015). Whilst binding to cognate antigen is the hallmark of antibody function, engagement of cell‐surface Fc receptors can activate immune cells to remove or neutralize antibody‐coated targets via processes known collectively as antibody effector functions (Desjarlais and Lazar, 2011). There are six classical Fc receptors recognizing IgG: high‐affinity FcγRI (CD64) and low‐affinity FcγRIIa, FcγRIIb, FcγRIIc (CD32a‐c), FcγRIIIa and FcγRIIIb (CD16a and b); and their cooperation on immune cells surface allows for a tailored cellular response against the target. Interaction of the antibody Fc region with FcγRs is influenced by glycans attached to a highly conserved *N*‐glycosylation site within the Fc fragment, and their composition can influence binding affinity and receptor‐mediated activity (Jefferis, 2009b).

An important feature of IgG antibodies is their long serum half‐life, which extends their therapeutic and protective effect (Ward *et al*., 2015). Rapid clearance of IgG from the circulation is prevented by interaction with neonatal Fc receptor (FcRn), which protects IgG antibodies from endosomal degradation (Junghans and Anderson, 1996). FcRn is a membrane heterodimer protein, which belongs to the MHC class I molecular family (Burmeister *et al*., 1994). Recent studies have demonstrated that efficacy of antibody therapy correlates with serum half‐life and that engineering antibodies to prolong their lifespan in the blood extends their therapeutic effect (Ko *et al*., 2014; Zalevsky *et al*., 2010).

The importance of Fc receptor interactions was recently highlighted for broadly neutralizing antibodies (bNAbs) in protection against HIV infection (Gautam *et al*., 2016; Ward *et al*., 2015). Various studies suggest that bNAbs can control persistent viral reservoirs, raising the possibility of their use for treating infected individuals (Caskey *et al*., 2015; Chun *et al*., 2014). Protective and therapeutic effects of antibodies are largely dependent on the antibody Fc region, and were dramatically decreased when FcR‐binding activities were engineered out of the antibodies (Bournazos *et al*., 2014). The role of FcγRs in antibody‐mediated protection against HIV infection was further emphasized in the RV144 vaccine trial, where an estimated 30% of vaccinated individuals were protected from acquiring HIV. Detailed analysis revealed that the protective effect correlated with induction of IgG3 responses and high ADCC activity of the elicited antibodies (Chung *et al*., 2014).

One of the first broadly neutralizing HIV antibodies discovered using next‐generation methods, VRC01 (Wu *et al*., 2010), has rapidly become an important clinical candidate for HIV prevention and treatment (Ledgerwood *et al*., 2015). A single injection of VRC01 antibody provided 8‐week protection against HIV infection in macaques, which was extended to 14.5 weeks by improving its binding to FcRn (Gautam *et al*., 2016). Moreover, an engineered VRC01 demonstrated increased FcRn‐mediated delivery to gut mucosal tissue resulting in enhanced mucosal localization and improved protection against SHIV challenge (Ko *et al*., 2014). In humans, VRC01 has been the subject of numerous completed and current clinical trials (Bar *et al*., 2016; Crowell *et al*., 2019; Riddler *et al*., 2018), which all demonstrate that the antibody is safe and well‐tolerated. Whilst protective efficacy has not been prominent, it is widely agreed that monoclonal antibody monotherapy is insufficient and that combination therapy with at least two mAbs is a minimum requirement (Mendoza *et al*., 2018).

Plants are becoming established as a ‘low‐tech’, cost‐effective and scalable production system for biopharmaceuticals including monoclonal antibodies (mAbs) (Paul *et al*., 2013). Molecular pharming is particularly attractive in the field of HIV antibody therapeutics as the major limitation of antibody‐based anti‐HIV agents remains their high cost of manufacturing (Ma *et al*., 2013). Recently, the first GMP manufacturing licence for plant mAbs was awarded in Europe, which led to a first‐in‐man phase I clinical trial (Ma *et al*., 2015). The only significant difference between mAbs produced in plant and mammalian cells results from different glycosylation pathways leading to production of antibodies with different glycan composition. Plant glycoproteins are characterized by core α1,3‐fucose and β1,2‐xylose resides, and lack of galactose and sialic acid residues (Bosch *et al*., 2013), which was shown to have a negative impact on ADCVI (antibody‐dependent cell‐mediated virus inhibition) activity against HIV of some (Forthal *et al*., 2010), but not all (Rosenberg *et al*., 2013), plant‐produced antibodies.

mAb VRC01 expressed in plants demonstrated similar antigen binding and HIV neutralization activity to VRC01 produced in HEK cells (Rosenberg *et al*., 2013; Teh *et al*., 2014). However, plant‐derived VRC01 was cleared rapidly from the bloodstream in macaques (Rosenberg *et al*., 2013). The reason for the rapid clearance observed for some plant‐made antibodies is unknown, and results are inconsistent (Khoudi *et al*., 1999; Lee *et al*., 2013; Triguero *et al*., 2011). It has been suggested that distinct glycosylation of recombinant antibodies expressed in nonhuman systems might have an indirect effect on FcRn binding (Jefferis, 2009a), even though human glycosylation does not affect binding of IgG to FcRn directly, because the receptor does not come in close contact with the Fc glycans (Martin *et al*., 2001; Nezlin and Ghetie, 2004).

In this study, the primary objective was to perform a comprehensive analysis of the impact of glycosylation on mAb VRC01 interactions with Fc receptors. Also, other post‐translational modifications, like methionine oxidation, were assessed in the context of FcRn binding. A panel of engineered glycovariants of VRC01 were generated in *N. benthamiana*, and Fc‐gamma receptor binding was characterized by kinetics and affinity measurement as well as effector function. The plant‐derived antibodies were evaluated for interactions with FcRn.

## Results

### VRC01 antibody glycovariants produced in *N. benthamiana*

cDNA sequences encoding antibody heavy and light chains were cloned into a binary vector (Sainsbury *et al*., 2009) and transiently expressed in *N. benthamiana* using agroinfiltration (Kapila *et al*., 1997). Purified antibodies were subjected to glycan analysis by liquid chromatography–electrospray ionization‐mass spectrometry (LC‐ESI‐MS) to identify and quantify *N*‐glycan populations in the Fc region. Each glycovariant was produced in 2–3 batches, and successful glycoengineering of extra batches was confirmed by immunoblotting (data not shown). Mass spectra of tryptic glycopeptides revealed that the main structure on VRC01 expressed transiently in WT *N. benthamiana* (VRC01_WT_) is a typical plant glycan with β1,2‐xylose and core α1,3‐fucose residues (GnGnXF^3^), accounting for 54.1% of the entire population (Table 1, Figure S1). A high proportion of VRC01_WT_ heavy chains (36%) appeared to be unglycosylated, and there were also minor populations of incompletely processed glycans. VRC01 expressed in the ΔXT/FT plant line (VRC01_ΔXF_), where β1,2‐xylosylation and core α1,3‐fucosylation had been silenced (Strasser *et al*., 2008), demonstrated a homogenous glycosylation pattern with a dominant structure GnGn (74.6%). No xylosylated or fucosylated glycan structures were detected, and in this case, 21.2% of the heavy chains were unglycosylated. To further modify the plant *N*‐glycans by addition of terminal β1,4‐galactose, VRC01 was co‐expressed with a human β1,4‐galactosyltransferase targeted to the late Golgi compartment, in the ΔXT/FT plant line (VRC01_Gal_). This resulted in heterogeneous glycosylation of the heavy chains, with several structures (approx. 50%) carrying at least one galactose residue. As in VRC01_∆XF_, no xylose and fucose residues could be detected in the VRC01_Gal_ heavy chain. Almost 30% of VRC01_Gal_ heavy chains were unglycosylated.

**Table 1:** Percentage composition of *N*‐glycan populations on the heavy chains of different VRC01 glycovariants

VRC01 HEK		VRC01 WT		VRC01 ∆XF		VRC01 Gal	
GnGnF^6^	70.5%	GnGnXF^3^	54.1%	GnGn	74.6%	unglyc.	29.4%
GnAF^6^	17.2%	unglyc.	35.9%	unglyc.	21.1%	MA + Man4Gn	28.7%
**unglyc.**	**3.1%**	**MGnXF** ^**3**^	**3.7%**	**MGn**	**4.3%**	Man4A + Man5Gn	10.8%
**MGnF** ^**6**^	**2.7%**	**MMXF** ^**3**^	**2.5%**			Man5A	9.9%
**GnAF** ^**6**^ **bi**	**2.4%**	**Man8**	**1.2%**			MGn	9.3%
**AAF** ^**6**^	**1.9%**	**Man9**	**1.0%**			AA	7.8%
**Man5**	**1.2%**	**Man7**	**0.7%**			**GnGn**	**3.3%**
**UGnF** ^**6**^	**0.9%**	**Man5**	**0.7%**			**MM**	**0.9%**
		**Man6**	**0.4%**				

Structures and abbreviations of *N*‐glycans which constitute <5% of the total population have been formatted in bold. Unglyc.–unglycosylated heavy chain. Abbreviations according to the ProGlycAn nomenclature (http://www.proglycan.com/protein-glycosylation-analysis/nomenclature).

The glycosylation profile of VRC01 antibody derived from HEK cells was also analysed (VRC01_HEK_), revealing a heterogeneous pattern with the most prevalent structure being GnGnF^6^ (70.5%). Galactosylated structures, but not sialylated, were also present. 3.1% of the heavy chains were unglycosylated. Finally, a plant‐produced VRC01 variant lacking Fc *N*‐glycans was created by site‐directed mutagenesis (VRC01_aglyco_). Disruption of the *N*‐glycosylation site by N_297_S mutation was verified through DNA sequencing, and the absence of *N*‐glycans on the heavy and light chains was further indicated at the protein level by SDS‐PAGE of antibodies analysed under reducing conditions (data not shown).

### Binding kinetics and affinity of VRC01 to FcγRI and FcγRIIIa

**Figure 1:**
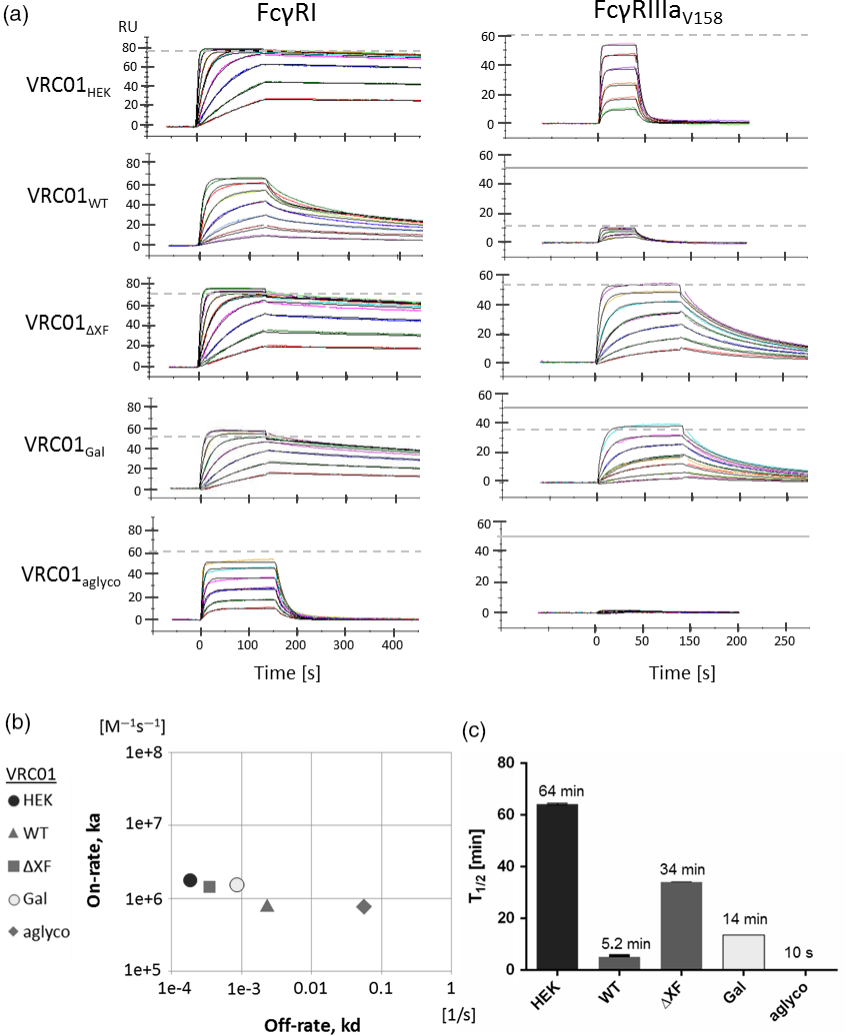
Interaction of VRC01 glycovariants with recombinant human FcγRI and FcγRIIIa. (a) SPR sensorgrams illustrate binding and dissociation of antibody–receptor complexes. Data were analysed with a 1 : 1 binding interaction model for FcγRI (except VRC01 _WT_ ; see text for details) and a two‐state reaction interaction model for FcγRIIIa. Dashed line denotes maximum response upon saturation. Theoretical saturation level is displayed separately as a solid line if it differs or could not be extrapolated from the data. Receptor concentrations used: FcγRI at 2–240 nm (15–480 nm for VRC01_aglyco_), and FcγRIIIa at 125–4000 nm for fucosylated antibodies and 12–800 nm for afucosylated ones. Data shown are from one experiment representative of at least two technical repeats. (b) Kinetic map for the interaction of antibodies with FcγRI based on association rate (on‐rate) and dissociation rate (off‐rate) constants. For VRC01 _WT_ , the kinetics parameters of the glycosylated portion were used. (c) Half‐life of the antibody–FcγRI complexes as calculated from the dissociation rate constants.

Binding kinetics and affinities of mAb Fc interactions with FcγRs were performed using surface plasmon resonance. Each VRC01 glycovariant was individually captured onto a protein A‐coated sensor chip, and recombinant human FcγRI ectodomains were injected over the antibody surface at a range of concentrations. Sensorgrams were analysed with a 1 : 1 binding model except for VRC01_WT_ where a modified version of the heterogeneous ligand binding model was used to fit the biphasic binding behaviour of the unglycosylated and glycosylated mix in VRC01_WT_ (Figure 1a). The model was created by incorporating kinetic parameters for unglycosylated VRC01 to the heterogeneous binding model to cover the unglycosylated portion of VRC01_WT_ and thus to extract kinetics for the glycosylated portion of VRC01_WT_.VRC01_Gal_ was also tested with both this heterogeneous binding model that assumes an aglycosylated portion of VRC01 and the standard 1 : 1 binding model (Figure S2). The same kinetics for galactosylated VRC01 were obtained regardless of the model choice—the aglycosylated portion of VRC01_Gal_ was recognized and discarded by the 1 : 1 binding model as artefact. Accordingly, the simplest model (usually 1 : 1 binding kinetics) was always chosen. Chi‐squared values were assessed for all kinetics analysis, and data are only reported when deviation of the fit was <5% *R*_max_.

VRC01HEK had the highest affinity to FcγRI with a dissociation constant (KD) of 98 pM, and VRC01ΔXF and VRC01Gal had KDs of 247 pM and 558 pM, respectively (Figure 3). VRC01_WT_ formed significantly less stable complexes with the receptor (KD = 3 nm), and removing Fc glycosylation reduced affinity to FcγRI by another order of magnitude (71 nm, VRC01_aglyco_) and caused immediate dissociation of the antibody–receptor complex (Figure 1a). Kinetic analysis revealed that changes in glycosylation mainly affected the dissociation rate constant, without influencing the association rate constant significantly (Figure 1b). As a consequence, glycosylation altered stability of antibody–FcγRI complexes, reducing their half‐life from about 1 h to 5 min for typical plant *N*‐glycans, and to only seconds for unglycosylated antibodies (Figure 1c).

To study binding to FcγRIIIa, a polymorphic variant with high affinity to IgG1, FcγRIIIa‐V158 (Ravetch and Perussia, 1989), was selected. Previous studies demonstrated that the interaction does not follow the 1 : 1 binding model despite 1 : 1 stoichiometry (Frostell, 2015), probably due to conformational change that may occur during the binding event (Hayes *et al*., 2014). Three‐dimensional structures of the FcγRIIIa–Fc complex suggest that the receptor makes close contact with one arm of the heavy chain first and then locks its position in between the two chains (Ferrara *et al*., 2011). For these reasons, the binding sensorgrams were analysed using the two‐state reaction interaction model. These revealed that VRC01_HEK_ binds FcγRIIIa with rapid on‐ and off‐rates (Figure 1a). The affinity was 620 nm (Figure 3). In contrast, VRC01_ΔXF_ and VRC01_Gal_ formed more stable complexes, primarily through slower off‐rates, resulting in approximately one order of magnitude higher affinity (32.6 and 59 nm, respectively). Additionally, for VRC01_Gal_ a 28% reduction in saturation level (*R*_max_) was observed, indicating that a proportion of molecules are inactive for FcγRIIIa binding. This correlated with 29.4% aglycosylation in VRC01_Gal_ sample that was detected by LC‐ESI‐MS (Table 1). Indeed, when *N*‐glycans were absent, the unglycosylated antibodies (VRC01_aglyco_) could not engage the receptor at any of the tested concentrations (Figure 1c). VRC01_WT_ exhibited an interesting binding pattern to FcγRIIIa, which is characterized by two aspects. Firstly, the binding responses saturated at 20% of the expected level, indicating that about 80% of the VRC01_WT_ molecules cannot engage FcγRIIIa at all. Secondly, the remaining 20% of VRC01_WT_ antibodies had a slightly higher affinity to FcγRIIIa than VRC01_HEK_, estimated to be 430 nm.

### Affinity to FcγRIIa and FcγRIIb

Implications of antibody glycovariants were also analysed for two low‐affinity FcγRs, FcγRIIa and FcγRIIb. For this study, the more prevalent polymorphic variant of FcγRIIa (R131) was selected (Osborne *et al*., 1994; Warmerdam *et al*., 1990).

**Figure 2:**
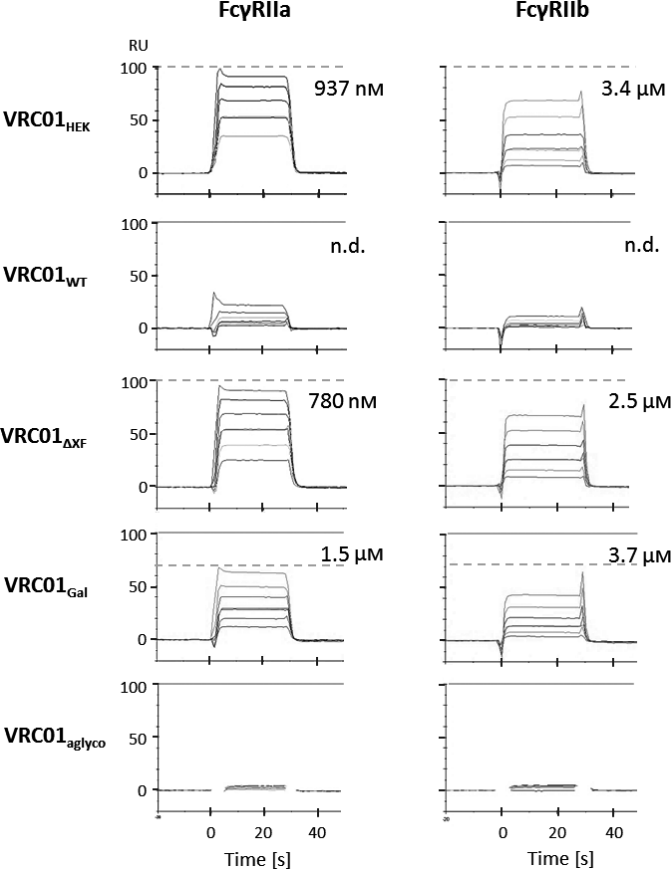
Affinity of VRC01 glycovariants to the human FcγRIIa and FcγRIIb. SPR sensorgrams show binding responses at the injection of different receptor concentrations (0.5–8 μm) over the Protein A‐captured antibodies. Dissociation constants are calculated from steady‐state responses at a dynamic equilibrium of complex formation. Maximum response at the saturation level is marked with dashed horizontal lines. When the experimentally measured response at saturation does not overlap with expected response, theoretical saturation levels are indicated with solid horizontal lines. Data shown are from one experiment representative of at least two technical repeats.

Steady‐state affinity measurements revealed that VRC01_HEK_ binds FcγRIIa with an affinity of 937 nm (Figure 2; steady‐state graph data are shown in Figure S3). Binding of the plant‐produced VRC01_WT_ was too low to measure under the assay conditions, but the affinity of the interaction appears to be lower than 7 μm. Engineering Fc glycosylation restored binding of plant‐made antibodies to FcγRIIa, as demonstrated by VRC01_ΔXF_ and VRC01_Gal_ (affinities 780 nm and 1.5 μm, respectively). As with FcγRIIIa, the VRC01_Gal_ preparation appears to contain approximately 29% inactive molecules, as indicated by a mismatch between the experimental and theoretical *R*_max_. This observation correlates with the extent of aglycosylation measured by mass spectrometry (Table 1).

Interaction of the VRC01 glycovariants with FcγRIIb exhibited a similar binding pattern as for FcγRIIa. Plant‐made VRC01_WT_ had an affinity lower than 7 μm, which was improved in the glycoengineered variants VRC01_ΔXF_ and VRC01_Gal_ (2.5 and 3.7 μm, respectively), reaching a similar level to VRC01_HEK_ (3.4 μm) (Figure 2; steady‐state graph data are shown in Figure S4). VRC01_aglyco_ did not bind FcγRIIa and FcγRIIb at any concentration tested, indicating that unglycosylated antibodies are not able to engage these receptors.

**Figure 3:**
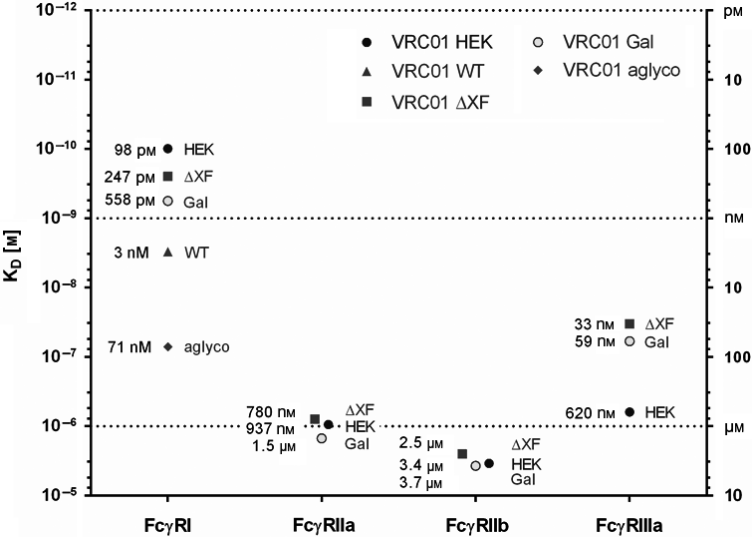
Affinity of VRC01 glycovariants to human FcγRs. The graph summarizes the affinity values (K_D_ ) for the interaction of different glycovariants with human FcγRI, FcγRIIa, FcγRIIb and FcγRIIIa.

The relative affinities for all antibodies against Fcγ receptors, measured by SPR, are summarized in Figure 3.

### Binding to cell‐surface FcγRs

**Figure 4:**
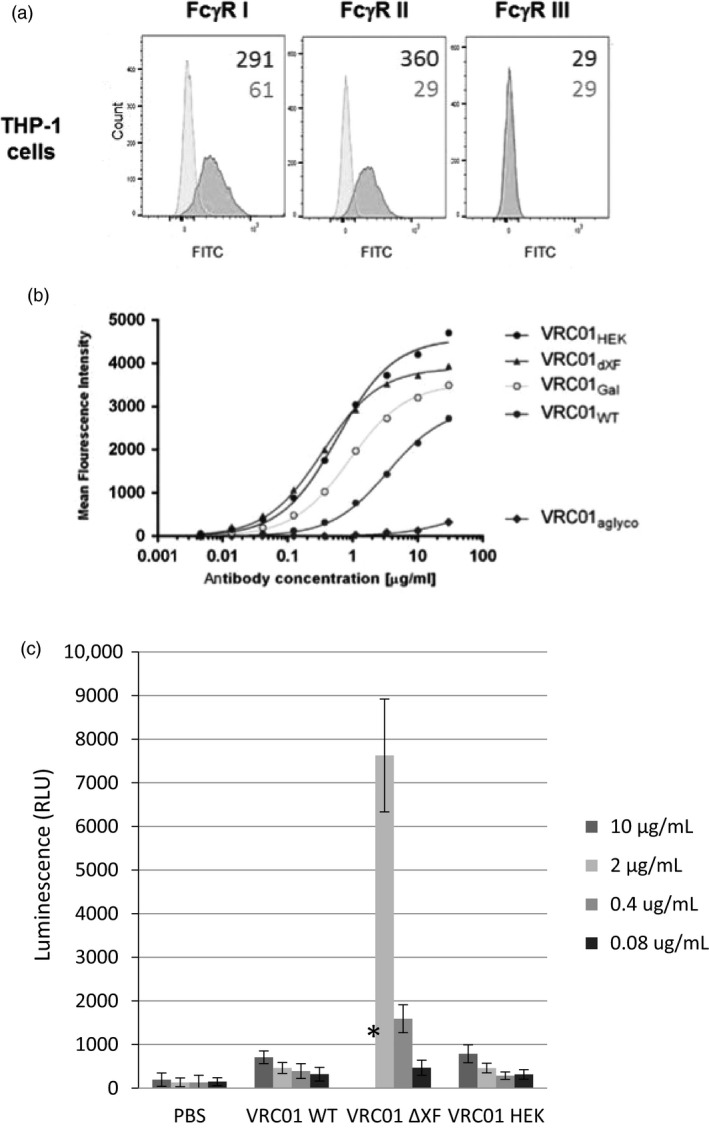
Binding of monomeric VRC01 glycovariants to cell‐surface Fc receptors on THP‐1 monocytes. (a) Flow cytometric analysis with FITC‐conjugated antibodies specific to FcγRI, FcγRII or FcγRIII on the surface of THP‐1 cells (red histograms) as compared to the isotype controls (blue histograms). The numbers indicate mean fluorescent intensity (MFI) of the populations. (b) Binding of serially diluted VRC01 antibodies to THP‐1 cells was detected with a FITC‐labelled F(ab’)_2_ fragment against human antibodies using flow cytometry. MFI responses were corrected by subtracting background response levels from cells incubated with the FITC‐labelled F(ab’)_2_ fragment only (no VRC01). Nonlinear regression curve fit was applied. The results represent one of two biological repeats. (c) FcγRIIIa activation by VRC01 _WT_ , VRC01_Δ_ _XF_ and VRC01 _HEK_ was demonstrated by luciferase activity of ADCC Bioassay Reporter cells. Antibodies were pre‐incubated with HIV‐1 UG37 gp140 before effector cells (Jurkat NFAT‐*luc*) were added. Luciferase activity was the average result of three independent experimental repeats. Error bars equals ± SD and PBS was used as negative control. * VRC01_Δ_ _XF_ at 10 μg/mL was omitted because the readings exceeded the range of the assay.

Binding of VRC01 glycovariants to FcγRs was further analysed in a cellular context, using THP‐1 monocytes (Tsuchiya *et al*., 1980). Flow cytometric analysis demonstrated that these cells express FcγRI and FcγRII receptors on the surface, but no FcγRIII could be observed (Figure 4a). The cells were incubated with VRC01 antibodies, and binding was detected with specific antibody fragments conjugated to FITC fluorochrome. All antibodies except VRC01_aglyco_ demonstrated binding to the cells (Figure 4b). However, VRC01_WT_ did not engage the cell‐surface FcγRs as effectively as VRC01_HEK_. Engineering plant glycosylation improved binding of the plant‐made antibodies to cells (VRC01_ΔXF_ and VRC01_Gal_). The observations are consistent with the binding results obtained in the SPR‐based assays.

To demonstrate a functional impact of increasing binding affinity to FcγRIIIa, an ADCC reporter bioassay was used (Figure 4c). Dilutions of VRC01_WT_, VRC01_ΔXF_ and VRC01_HEK_ were pre‐incubated with HIV‐1 gp140, before incubating with engineered Jurkat effector cells (ADCC Bioassay Effector cells). In this assay, FcγRIIIa activation is directly proportional to the luciferase activity in the effector cells. FcγRIIIa activation induced by VRC01_WT_ and VRC01_HEK_ was similar. However, FcγRIIIa activation by VRC01_ΔXF_ was approximately 25‐fold higher. This is consistent with the results of the FcγRIIIa analysis described above as well as other studies (Forthal *et al*., 2010; Junttila *et al*., 2010; Zeitlin *et al*., 2011). Luciferase activity of control samples with no antibody (gp140 + cells only) or no gp140 (mAb + cells only) was not significantly different to PBS (data not shown).

### Binding to FcRn

FcRn is an important receptor that can influence and modulate the course of antibody‐based therapy. Although the exact binding mechanism *in vivo* is still unclear, recent studies provide strong evidence that each IgG heavy chain can bind one FcRn molecule independently with identical affinity (Abdiche *et al*., 2015; Oganesyan *et al*., 2014). The resulting 2 : 1 FcRn–IgG complex is then characterized by stronger interaction due to avidity (Tesar *et al*., 2006).

**Figure 5:**
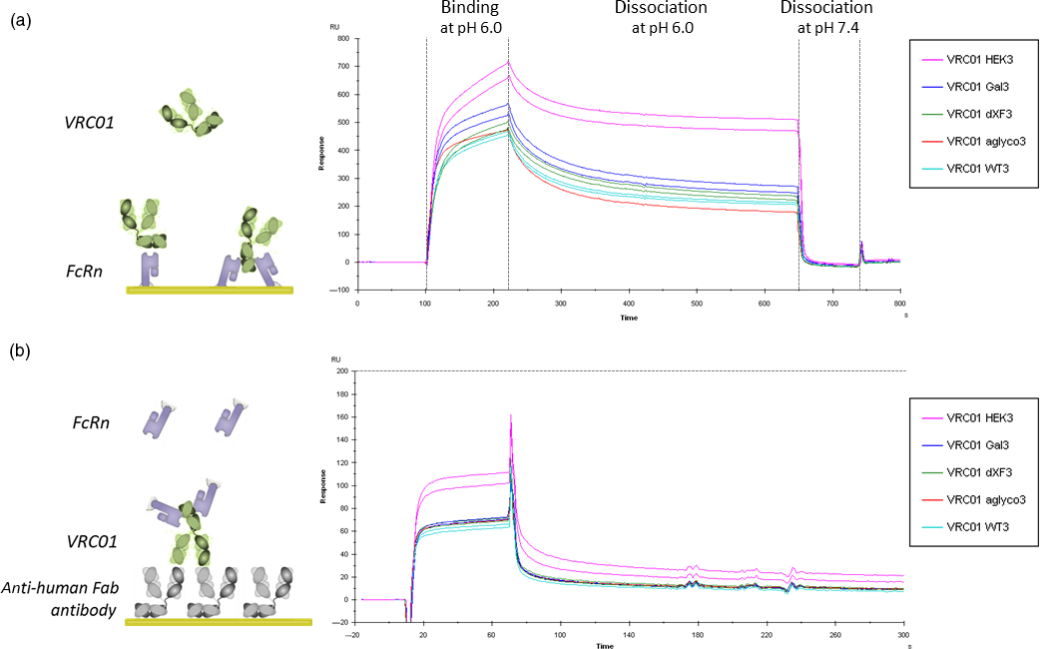
Binding analysis of VRC01 glycovariants to FcRn. (a) Human recombinant FcRn ectodomains were covalently immobilized on the CM5 sensor chip surface and different VRC01 glycovariants were injected at 200 nm concentration in duplicate. Data shown are from one experiment representative of at least two technical repeats. Binding and dissociation of different antibodies was observed at pH 6.0, and the FcRn surface was regenerated with PBS (pH 7.4). (b) Each VRC01 glycovariant was captured onto the anti‐human Fab surface to the same level and tested for binding to 200 nm FcRn. Sensorgrams represent binding and dissociation at pH 6.0 for two independent repeats. Maximum binding level is marked with a dashed horizontal line.

SPR analysis was performed in two assay orientations. FcRn was either immobilized on the sensor chip surface allowing antibodies to slot between two receptors, resulting in strong avidity interaction, or they were injected in solution over an antibody‐coated surface allowing for independent interaction with each antibody arm. Due to the pH‐dependent nature of the interaction, where antibodies engage the receptor in acidic conditions of the endosome and are released at physiological pH of the blood (Vaughn and Bjorkman, 1998), the binding in this assay was measured at pH 6.0. In the first strategy, different VRC01 glycovariants were injected at 200 nm in duplicate (Figure 5a). The sensorgrams show that VRC01_HEK_ generates the highest binding responses, indicating higher affinity, followed by all plant‐produced variants, which have comparable binding levels, including unglycosylated VRC01. Formation of stable multivalent complexes was observed, as characterized by very slow dissociation curves. All the complexes dissociated efficiently at physiological pH 7.4. In the second, avidity‐free assay orientation, soluble monomeric FcRn was injected at 200 nm in duplicate (Figure 5b). Again, VRC01_HEK_ generated the highest binding responses and FcRn bound all the plant‐produced variants similarly, to a level of around 2/3 of VRC01_HEK_. The observed difference suggests a higher affinity of VRC01_HEK_ to FcRn; however, the presence of partially degraded antibodies in plant samples is commonly found (Hehle *et al*., 2011) and could also cause a decrease in observed binding levels. Therefore, affinity values for all VRC01 glycovariants to recombinant human FcRn was measured in a steady‐state affinity assay. Briefly, each antibody was captured to the same level onto an anti‐human Fab surface, followed by injection of FcRn at a range of concentrations (Figure S5). The steady‐state affinity measurements revealed that all glycoforms have a fairly similar affinity to FcRn, with VRC01_HEK_ exhibiting slightly higher affinity (199 nm) than the plant‐produced variants (272–310 nm). The difference is unlikely to result from different plant glycosylation, given that neither glycoengineering (VRC01_ΔXF_ and VRC01_Gal_) nor removing the Fc glycan (VRC01_aglyco_) affected antibody affinity to the receptor significantly. Another possibility might be antibody oxidation, which has been previously reported to reduce antibody affinity to FcRn (Bertolotti‐Ciarlet *et al*., 2009; Pan *et al*., 2009), and this was investigated in the next experiment.

### Methionine oxidation

It was previously observed that oxidation of two methionine residues, Met252 and Met428, on the antibody heavy chain can result in up to 10‐fold reduced affinity to FcRn (Bertolotti‐Ciarlet *et al*., 2009; Gao *et al*., 2015). The plant‐made VRC01 samples, together with a new VRC01_HEK_ batch, were therefore analysed to measure percentage oxidation of these two methionine residues.

Heavy chains were separated by gel electrophoresis and enzymatically digested to corresponding peptides containing Met252 or Met428 residues: DTLM^252^ISR and SCSVM^428^HEALHNHY. The relative percentage oxidation was calculated from MS spectra, by comparing a proportion of the peak heights corresponding to an oxidized versus nonoxidized peptide containing either the Met252 or Met428 residues. The sequence identity of oxidized and nonoxidized peptides (both DTLM^252^ISR and SCSVM^428^HEALHNHY), as well as formation of methionine sulfoxide (MetOx), was further confirmed by MS‐MS (data not shown).

**Figure 6:**
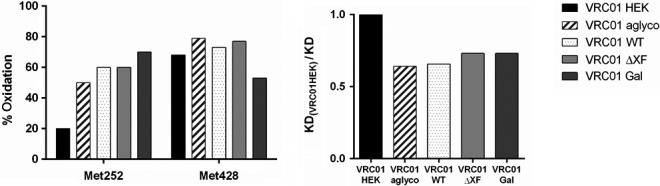
Methionine oxidation of VRC01 glycovariants. (a) Percentage oxidation of Met252 and Met428 on antibody heavy chains, as determined by mass spectrometry. All plant‐made VRC01 glycovariants were produced at the same time under the same conditions and stored in nonoxidizing atmosphere. Data shown are from one experiment representative of at three biological repeats. (b) Relative affinity of plant‐made VRC01 glycovariants to FcRn compared to VRC01 _HEK_ . Values obtained from SPR analysis.

Mass spectrometry analysis of methionine oxidation revealed that all the plant‐produced VRC01 antibodies contained significantly more methionine sulfoxide in the Met252 position (50%–70%) as compared to VRC01_HEK_ (20%) (Figure 6). Met428 displayed a different oxidation pattern than Met252, with VRC01_HEK_ and all plant antibody glycovariants having similar oxidation levels 50%–76%.

Four separate batches of VRC01_WT_ were subsequently produced to investigate the possibility of controlling Met252 and Met428 oxidation during plant antibody extraction, purification and storage. The batches of VRC01_WT_ were prepared, with and without the addition of an antioxidant during antibody extraction, and stored under nitrogen gas or not. Mass spectrometry analysis of methionine oxidation was performed as above. There was no significant difference in Met252 or Met428 oxidation between any of the downstream processing strategies (data not shown), suggesting that methionine oxidation is likely to take place *in planta*, rather than during antibody extraction and storage.

## Discussion

Four activating receptors, FcγRI, FcγRIIa, FcγRIIc and FcγRIIIa, and one inhibitory receptor, FcγRIIb (Ravetch, 2010), are differentially distributed on immune cells. They can be found on all leucocytes and platelets except T cells (Guilliams *et al*., 2014; Hogarth and Pietersz, 2012), and many cells have multiple FcγRs on their surface. Once bound to antigen, antibodies can mediate a range of immune functions through interaction with FcγR. Engagement of activating FcγRs can induce (i) antibody‐dependent cell‐mediated cytotoxicity (ADCC) or antibody‐dependent cell‐mediated phagocytosis (ADCP), which both result in direct killing of antibody‐opsonized cells, (ii) release of pro‐inflammatory mediators and cytokines, to attract other white blood cells to the infection site and activate them, and (iii) antigen endocytosis and presentation, to further enhance the immune response (reviewed in Desjarlais and Lazar, 2011).

In order to study the effect of plant *N*‐glycosylation on recombinant antibody Fc‐mediated binding activities, the broadly neutralizing HIV antibody, VRC01, was expressed in plants in different glycol formats. Our studies revealed that VRC01 with typical plant glycosylation had 30‐fold lower affinity to recombinant human FcγRI than the same antibody produced in HEK cells. Also, it had a limited ability to bind the low‐affinity receptors FcγRIIa, FcγRIIb and FcγRIIIa. Removal of Fc glycans by mutagenesis of Asn297 resulted in a complete loss of binding. However, engineering plant glycans by removal of core fucose and xylose significantly improved affinity of plant‐made VRC01 to FcγRI and restored binding activity to FcγRIIa and FcγRIIb. Moreover, affinity of fucose/xylose‐free antibodies to FcγRIIIa was 10–20 times enhanced as compared to the HEK cell‐derived VRC01. The impact of fucose removal on binding to FcγRIIIa was previously observed for antibodies generated in mammalian cell and plant‐based systems (Cox *et al*., 2006; Shields *et al*., 2002) and likely results from steric interference with glycans present in the binding site of FcγRIIIa (Ferrara *et al*., 2011). Extending the fucose‐ and xylose‐free antibodies with terminal β1,4‐galactose residues did not further improve their interaction with Fc‐gamma receptors. Cell‐based binding assays with THP‐1 monocytes confirmed the binding behaviour of different VRC01 glycovariants observed in the SPR analysis.

The binding of VRC01_WT_ to FcγRIIIa indicated that about 80% of antibody molecules did not engage the receptor. The remaining active proportion had an affinity of 430 nm, as compared to 620 nm for VRC01_HEK_. The inactive proportion correlates with the proportion of molecules either with no Fc glycans or containing typical plant GnGnXF^3^ structures. This suggests that not only unglycosylated antibodies, but also GnGnXF^3^ glycoforms might not be able to bind FcγRIIIa. The core fucose in plants is α1,3‐linked, rather than the α1,6‐link in mammalian systems, but the results suggest the differentially oriented fucose on plant‐produced antibodies still causes steric constraints in the FcγRIIIa binding site, abrogating the interaction. The active portion of VRC01_WT_ antibodies, which engages FcγRIIIa more effectively than VRC01_HEK_, might correspond to the non‐fully processed glycoforms that were found in the sample. These comprise oligomannose structures lacking core fucose and truncated complex glycans containing fucose. Thus, VRC01_WT_ antibody produced in plants appears to be a mixture of FcγRIIIa nonbinders and binders with different affinities, depending on the glycoform they carry. This variability may translate functionally into potentially different ADCC activities.

Nonglycosylation had a significant effect on antibody–receptor binding. Glycopeptide analysis revealed that all VRC01 antibody preparations contained a proportion of unglycosylated heavy chains. This was very low in the HEK‐derived VRC01 (<5%), but reached higher levels when the antibody was expressed in *N. benthamiana* (21%–35% depending on the glycoengineering strategy). Recombinant glycoproteins generated in plant systems are often fully glycosylated; however, some studies have previously reported partial aglycosylation of plant‐made antibodies (Jez *et al*., 2012; Schuster *et al*., 2007; Teh *et al*., 2014; Zeitlin *et al*., 2011). The reason for hypoglycosylation of some plant‐made antibodies is unknown, but seems to be antibody specific. A powerful strategy for enhancing glycosylation efficiency is the over‐/co‐expression of the oligosaccharyltransferase complex (OST), which is the central‐protein complex facilitating the *N*‐glycosylation of proteins in the lumen of the endoplasmic reticulum (Castilho *et al*., 2018).

Serum antibodies naturally demonstrate a high level of glycan heterogeneity, exhibiting as many as 30–40 different glycoforms (Nimmerjahn and Ravetch, 2008; Stadlmann *et al*., 2008). The functional relevance of such glycan heterogeneity is not fully understood; however, the glycoform profile may be skewed in pathology or in response to pathogens (Ackerman *et al*., 2013; Jefferis, 2012). This indicates that glycan heterogeneity might be important and antibodies with heterogeneous glycoprofile might actually be preferred over those with a single glycoform. However, a better understanding of the role of individual glycan residues is needed first, before we can start to consider how best to mimic a natural antibody response.

At present, therapeutic monoclonal antibodies are generally obtained from mammalian cell‐based production systems which usually generate a mixture of about 5–9 different glycoforms (Stadlmann *et al*., 2008). Given that individual glycoforms may have different functional activities, such a mixture may potentially contain antibody molecules with limited or no activity. In future, control of the glycosylation profile of monoclonal antibodies for therapeutic use may therefore need to be considered more carefully. An ultimate goal might be to develop a reproducible optimized mix of glycoforms for a specific application.

Attempts to isolate a single glycoform from a heterogeneous mixture of glycovariants by affinity chromatography have met with limited success (Bolton *et al*., 2013), and purification of a single glycoform is currently not feasible (Loos and Steinkellner, 2012). Several approaches have been undertaken to reduce antibody glycosylation heterogeneity, including use of glycan‐modifying enzymes in vitro (Thomann *et al*., 2015) and genetic modification (Piron *et al*., 2015). Plants offer an attractive solution for producing highly homogeneous antibodies and other proteins with defined glycosylation (Madeira *et al*., 2016; Montero‐Morales and Steinkellner, 2018). Although expression of antibodies in WT plants can yield a proportion of non‐fully processed glycans, glycoengineered plant lines usually produce highly homogeneous glycoforms (Ma *et al*., 2015a; Strasser *et al*., 2008, 2009). Here, VRC01 antibody generated in ΔXF *N. benthamiana* contained a single predominant glycoform, GnGn, accompanied by MGn (4.7%). Although LC‐ESI‐MS analysis suggested the presence of 20% of unglycosylated heavy chains, this could not be confirmed in Western blot or SPR analyses. Typically, inactivation of endogenous glycosyltransferases in plants results in over 80% purity of GnGn glycoforms (Castilho *et al*., 2015; Strasser *et al*., 2008). Galactosylated VRC01_Gal_ produced in this study by transient co‐expression of human galactosyltransferase was characterized by high heterogeneity; however, a recently developed plant transgenic platform is able to produce antibodies lacking core fucose and xylose where 90% of molecules carry galactosylated *N*‐glycans, including 60% of bi‐galactosylated structures (AA) (Schneider *et al*., 2015).

Antibody binding to neonatal Fc receptor is reported to be independent of Fc glycosylation (Nezlin and Ghetie, 2004), as FcRn engages residues present on the CH2–CH3 domain interface (Burmeister *et al*., 1994; Oganesyan *et al*., 2014), but a concern was raised that unusual glycan structures from nonmammalian protein expression systems may affect the binding indirectly(Jefferis, 2009a). Our SPR analysis confirmed that plant *N*‐glycosylation did not have an adverse effect on binding to FcRn. All plant VRC01 glycovariants had similar affinity to FcRn that was slightly lower than VRC01 from HEK cells. However, binding reduction correlated with increased oxidation levels of a methionine residue (Met252) present in the Fc region, which has previously been reported to have a negative impact on the IgG–FcRn interaction (Gao *et al*., 2015).

Protein oxidation in recombinant protein production is an important factor influencing stability and functionality of protein pharmaceuticals (Li *et al*., 1995; Pan *et al*., 2009). Susceptible amino acids, like His, Met, Cys, Tyr and Trp, can be oxidized by a number of factors at any stage of the production, extraction, purification and storage (Manning *et al*., 2010). Methionine residues are particularly sensitive to reactive oxygen species and can oxidize to methionine sulfoxide even in the presence of molecular oxygen. Different methionine residues can oxidize at different speeds, with surface‐exposed residues oxidizing faster, whereas those buried in a protein backbone oxidize more slowly (Griffiths and Cooney, 2002).

Extraction of recombinant proteins from plant tissues presents many challenges. Plant leaves are rich in polyphenols (Li *et al*., 2003), which, in response to tissue damage and exposure to air, are enzymatically oxidized to reactive semiquinone radicals and quinones, which further attack sensitive residues on proteins (Met, Cys, Trp) (Synge, 1975). Polyphenol oxidation can also occur during protein extraction from plants (Hurrell *et al*., 1982). It has also been reported that during extraction, methionine sulfoxide is formed (Pirie, 1975). Protection of recombinant proteins from oxidation during plant extraction has not been widely studied; however, general preventive procedures have been used, for example thiosulfate, citrate (Chargelegue *et al*., 2005), ascorbic acid or Gamborg's B5 vitamin solution (Miletic *et al*., 2015). Reducing polyphenol oxidation can be attempted by addition of chelating agents like EDTA, and sometimes sucrose is also included, to limit solvent accessibility (Miletic *et al*., 2015). In our study, oxidation of Met252 and Met 458 was unaffected by antioxidant in the antibody extraction buffer, or storage in nonoxidizing conditions after extraction. Although further studies are required, this suggests that antibody oxidation may occur at an earlier stage in antibody expression, pointing to the likelihood that antibody mutagenesis strategies (Dall'Acqua *et al*., 2002; Zalevsky *et al*., 2010) might prove more useful.

In conclusion, this is a comprehensive report of the impact of two post‐translational modifications affecting antibodies produced in plants, on their Fc effector functions. We demonstrated the importance of glycosylation for binding to high‐ and low‐affinity Fcγ receptors and the potential role of glycoengineering strategies in plants to produce antibodies with potent opsonization and ADCC activity. We extended our studies to formally demonstrate that plant glycosylation does not significantly affect binding of antibodies to FcRn receptor, but that oxidation of at least one methionine residue in the Fc region has an impact on FcRn affinity. Our results suggest that the reported accelerated clearance of some plant‐produced antibodies from the bloodstream (Ko *et al*., 2003; Rosenberg *et al*., 2015; Triguero *et al*., 2011) might be attributable to this and other clearing mechanisms.

## Materials and methods

### VRC01_HEK_ was obtained from the Centre for AIDS Reagents, UK

#### Site‐directed mutagenesis and gene cloning

VRC01 heavy‐ and light‐chain gene sequences were plant‐codon‐optimized for tobacco (provided by Mapp Biopharmaceutical, Inc.). The heavy‐chain sequences were mutagenized using the QuikChange^®^ (Stratagene) protocol and primers designed to replace the Asn codon with Ser at position 297. Transformed colonies were selected and plasmids were isolated using QIAprep^®^ Spin Miniprep Kit (Qiagen) and confirmed by sequencing. Both mutated and original sequences of VRC01 light and heavy chains were subcloned into the plant expression vector pEAQ‐HT‐DEST3 (Sainsbury *et al*., 2009) by LR recombination using the Gateway cloning system (Invitrogen). DH5α competent *E. coli* cells were transformed, and colonies containing pEAQ‐HT vectors with appropriate gene constructs were selected using kanamycin. Expression vectors were isolated by QIAprep^®^ Spin Miniprep Kit (Qiagen).

### Antibody expression in *N. benthamiana* and purification

Expression vectors pEAQ‐HT‐DEST3 containing heavy and light genes were introduced into two different *Agrobacterium tumefaciens* strains: GV3101:pMP90 was used for the heavy‐chain construct and LBA4404 for the light‐chain construct. Agrobacteria were grown overnight at 28 °C in YENB medium with 50 μg/mL rifampicin and appropriate antibiotics to select for helper plasmid and expression vector. For infiltration, bacteria were resuspended in 0.1 mm acetosyringone (Santa Cruz Biotechnology), 0.01 mm MES (Sigma, Poole, UK) and 0.01 mm MgCl2 (VWR International, UK) to a final OD_600_ of 0.2. Vacuum infiltration of *N. benthamiana* leaves was performed as previously described (Kapila *et al*., 1997), using 4‐week‐old plants and applying vacuum for 1 min at 100 mbar. To produce glycoengineered VRC01 antibodies (VRC01ΔXF), the *N. benthamiana* ΔXF plant line (Strasser *et al*., 2008) was used for agroinfiltration. To generate the galactosylated glycovariant (VRC01_Gal_), the ΔXF *N. benthamiana* plants were additionally co‐infiltrated with agrobacteria containing pST plasmid with human β1,4‐galactosyltransferase (GalT) (Strasser *et al*., 2009). Vacuum infiltrated leaves were harvested at 6 days postinfection. Antibodies were extracted in 100 mm Tris buffer (pH 8.0) and 150 mm NaCl and purified using a Protein A‐Agarose (Sigma) column, eluting antibodies with 0.1 m glycine–HCl (pH 2.7) into 1 m Tris–HCl (pH 9.0) neutralizing buffer. Purified samples were dialysed into PBS with Slide‐A‐Lyzer Cassettes (molecular cut‐off 3.5 kDa; Thermo Scientific, Waltham, MA, USA) and stored under nitrogen in glass HPLC tubes with septum (10 mm PP Screw Cap; Fisher Scientific, Loughborough, UK).

For cell assays, the concentration of full‐size antibodies was calculated using densitometry analysis of identified IgG band on Coomassie‐stained SDS‐PAGE gels. Standard curves for known concentrations of bovine serum albumin and another plant‐derived IgG antibody (P2G12) were generated using the BCA Protein assay, and VRC01 concentration values were interpolated from nonlinear regression fit using second‐order polynomial (quadratic) equation.

In the experiment to control antibody oxidation during processing and storage, VRC01_WT_ was expressed in the same way except that antibodies were extracted using PBS (pH 7.4) with or without 10 mm sodium metabisulfite.

### LC‐ESI‐MS confirmation of glycoengineering

Following separation by SDS‐PAGE under reducing conditions, heavy‐chain bands were excised, S‐alkylated with iodoacetamide and digested with sequencing‐grade modified trypsin (Promega, Southampton, UK). Fragments were eluted from the gel with 50% (vol/vol) acetonitrile and loaded on a BioBasic C18 column (BioBasic‐18, 150 × 0.32 mm, 5 μm; Thermo Scientific) using 65 mm ammonium formate buffer as the aqueous solvent. A gradient from 5% B (B: 100% acetonitrile) to 32% B in 45 min was applied at a flow rate of 6 μL/min, followed by a 15‐min gradient from 32% B to 75% B that facilitates elution of large peptides. Detection was performed with a QTOF MS (Bruker maXis 4G) equipped with the standard ESI source in positive‐ion, data‐dependent mode (=switching to MSMS mode for eluting peaks). MS scans were recorded (range: 300–2000 Da), and the 6 highest peaks were selected for fragmentation. Instrument calibration was performed using an ESI calibration mixture (Agilent, Santa Clara, CA, USA). Results were evaluated using Data Analysis 4.0 (Bruker). Co‐eluting glycopeptide peaks were identified by their exact mass.

### FcγR binding kinetics and affinity measurements

SPR assays were performed using a Biacore^™^ X100 instrument (GE Healthcare, Little Chalfont, UK). First, a protein A sensor chip was prepared by immobilizing recombinant protein A (Sigma) in both flow cells of a CM5 sensor chip to 5000 RU using the Amine Coupling Kit (GE Healthcare). VRC01 samples were diluted in HBS‐EP+ buffer and captured onto the Protein A surface (flow cell 2) to the levels around 240RU (for FcγRI analysis) or 330RU (for FcγRIIIa) or 680RU (for FcγRIIa and FcγRIIb). Recombinant human FcγR ectodomains (R&D) were injected over both flow cells at 25 °C for 135 s at 40 μL/min (FcγRI), 40 s at 50 μL/min (FcγRIIIa when tested against fucosylated antibodies), 90 s at 40 μL/min (FcγRIIIa when tested against afucosylated antibodies) or 30 s at 45 μL/min (FcγRIIa and b). The FcγR–VRC01 complexes were removed with two 90‐s injections of 10 mm glycine–HCl (pH 1.5). If the regeneration was not fully successful, the first injection was replaced with 10 mm glycine–HCl–3.5M MgCl_2_ (pH 1.5) buffer. Receptors were analysed at the following concentrations: FcγRI—2–240 nm (480 nm for VRC01aglyco); FcγRIIIa—0.125–4 μm (12–800 nm for afucosylated antibodies VRC01_ΔXF_ and VRC01_Gal_); and FcγRIIa and FcγRIIb—0.5–8 μm. Half‐life of antibody–receptor complexes was calculated from dissociation rate constants according to the formula: *T*_1/2_ = ln2/*k*_off_.

### Surface expression of FcγRs on THP‐1 cells

The THP‐1 cell line (ATCC) was grown in RPMI culture medium at 37 °C and 5% CO_2_ atmosphere. The cells were harvested by centrifugation (300 RCF, 5 min) and concentrated to 2 × 10^6^ cells/mL in PBS with 2.5% BSA and 0.1% sodium azide (FACS buffer). To determine FcγRI expression, the cells were incubated with 10 μg/mL mouse anti‐human FcγRI antiserum (R&D) for 1 h on ice. Mouse IgG1 (Sigma) was used as an isotype control. The cells were washed twice with the FACS buffer by centrifugation at 300 RCF for 5 min and incubated with 1 : 100 FITC‐conjugated anti‐mouse immunoglobulins antibody (The Binding Site). For FcγRII and FcγRIII expression, the cells were incubated with 0.25 μg/test of FcγRII‐FITC or FcγRIII‐FITC antibody (eBioscience) for 1 h on ice. Mouse IgG1‐FITC (BD Biosciences) was used as an isotype control. The cells were washed twice, and fluorescence intensity was measured by BD FACSCanto™ II Flow Cytometer (BD Biosciences) using BD FACSDiva™ software. The data were analysed using FlowJo V10.1 software.

### VRC01 binding to cell receptors

THP‐1 cells were harvested by centrifugation (300 RCF, 5 min) and concentrated to 5 × 10^6^ cells/mL in FACS buffer. The cells were incubated with serially diluted VRC01 samples for 1 h on ice, and excess antibodies were washed off twice with FACS buffer by centrifugation at 300 RCF for 5 min. Binding of VRC01 samples to THP‐1 cells was detected with 1 : 200 FITC‐conjugated F(ab’)_2_ fragment against human Ig light and heavy chains (Jackson ImmunoResearch Laboratories, Inc.) by 20‐min incubation on ice. The cells were washed twice, and fluorescent intensity was measured by BD FACSCanto^™^ II Flow Cytometer (BD Biosciences). Mean fluorescent intensity (MFI) values were derived by subtracting MFI of THP‐1 cell populations treated with the F(ab’)_2_ fragment only. Binding curves were created using nonlinear curve fit (One site – Specific binding) with GraphPad Prism 6 software.

### FcγRIIIa activation reporter assay

The ADCC Reporter Bioassay was performed according to the manufacturer's protocol (Promega), using soluble HIV‐1 UG37 gp140 (CFAR, UK) instead of target cells. 0.08, 0.4, 2 and 10 μg/mL antibody was pre‐incubated with free HIV‐1 gp140 (5 μg/mL) for 1 h. ADCC Bioassay Effector cells (Jurkat cells stably expressing the high‐affinity (V158) FcγRIIIa receptor and an NFAT response element driving expression of firefly luciferase) were added at 1.5 × 10^5^ cells/well, and the assay plates were incubated at 37 °C and 5% CO_2_ for 24 h. Bio‐Glo^™^ luciferase assay reagent was added and luminescence measured in a GloMax 96 plate reader (Promega). In this assay, luciferase activity in the effector cells is directly proportional to FcγRIIIa activation.

### FcRn binding analysis

For binding analysis, two strategies were employed. Firstly, recombinant human FcRn ectodomain (SinoBiological Inc., Beijing, China) was diluted in 10 mm acetate buffer (pH 4.5) and immobilized on the CM5 sensor chip surface to 1000 RU by amine coupling (Amine Coupling Kit; GE Healthcare). Reference surface was ‘blank‐immobilized’ by activation with NHS/EDC and blocking with 1 m ethanolamine–HCl (pH 8.5). Binding analysis was performed in PBS‐P+ buffer (PBS 0.05% Tween‐20) (pH 6.0). To minimize differences in pH between different samples, VRC01 antibodies were prediluted to 1200 nm in PBS (pH 7.4) and then injected at 100 nm in PBS‐P+ (pH 6.0) for 120 s contact time and 120 s dissociation time at a flow rate of 40 μL/min. Samples were run in duplicate, and the surface was regenerated with a 60‐s injection of PBS (pH 7.4).

In the second strategy, anti‐human Fab antibodies (Fab Capture Kit) were diluted to 20 μg/mL in 10 mm acetate buffer (pH 5.0) and immobilized on a CM5 sensor chip to 6000 RU in both flow cells, by amine coupling. VRC01 antibodies were then captured in flow cell two only. Recombinant human FcRn (SinoBiological) was injected over the antibody surface at 100 nm for 60 s at a flow rate of 30 μL/min with 90 s dissociation time. All samples were run in PBS‐P+ (pH 6.0) running buffer and were analysed in duplicate. The chip surface was regenerated with 10 mm glycine–HCl (pH 2.1).

For FcRn affinity measurements, VRC01 antibodies were captured on the anti‐Fab surface. Recombinant human FcRn ectodomains (SinoBiological) were injected over the antibody surface at a range of concentrations (12.5–800 nm in PBS‐P+, pH 6.0) for 40 s at a flow rate of 30 μL/min and with 60 s dissociation time. After each injection, the antibody surface was regenerated with PBS (pH 7.4). 10 mm glycine–HCl (pH 2.1) was used to remove antibodies from the anti‐Fab surface. Binding responses at 10 s after injection from the double‐referenced sensorgrams were selected for affinity evaluation using a steady‐state affinity model (Biacore^™^ X100 Evaluation Software).

### Methionine oxidation

The heavy and light chains of purified antibody samples were separated by 12% Bis‐Tris NuPAGE^®^ polyacrylamide gel electrophoresis. Heavy chains were excised from the gel and washed three times for 15 min at RT with 0.1 m NH_4_HCO_3_ (pH 8) and 100% CH_3_CN (v:v). The samples were dried in a SpeedVac centrifuge (Thermo Fisher, Waltham, MA, USA). After reduction with 0.1 m dithiothreitol (DTT) for 45 min at 56 °C and alkylation with 55 mm iodoacetamide (IAA) performed in 0.1 m NH_4_HCO_3_ (pH 8) for 30 min at room temperature in the dark, 3 μg of proteomic‐grade trypsin was added (Promega) per sample and placed at 4 °C for 30 min prior to overnight incubation at 37 °C. The trypsin was inactivated by heating for 5 min at 100 °C. The samples were cooled to RT and digested with 5 μg of chymotrypsin (Sigma) for 4 h at 37 °C. The chymotrypsin was inactivated by heating 5 min at 100 °C. After the protease digestions, the gel pieces were incubated in solutions of 50% CH_3_CN, 5% formic acid, 0.1 m NH_4_HCO_3_ and 100% CH_3_CN and finally in 5% formic acid to extract the resulting peptide and glycopeptide mixture. The sample was dried before further analysis by QTOF Agilent Technologies LC‐Chip MS for high‐sensitivity nanospray LC‐MS (Agilent Technologies) as previously reported (Vanier *et al*., 2015).

## Author contribution statement

SS, RA, JM, MP and AT made substantial contributions to the conception and design of the study, to the organization of the conduct of the study, to carrying out the study and to analysis and interpretation of study data. MG, JV and MB carried out methionine oxidation studies, and analysed and interpreted data. FA and AC carried out glycosylation studies, and analysed and interpreted the data. All authors helped draft the output and contributed intellectual content.

## Supporting information


**Figure S1** Fc glycosylation profiles of the VRC01 antibody (HEK) and its plant‐made glycovariants (WT, ∆XF, Gal).
**Figure S2** Comparison between 1 : 1 binding interaction and ‘heterogeneous antibody with aglycosylation’ modelling for VRC01_Gal_ antibody binding to FcγRI.
**Figure S3** Steady‐state affinity measurements of different VRC01 glycovariants binding FcγRIIa.
**Figure S4** Steady‐state affinity measurements of different VRC01 glycovariants binding FcγRIIb.
**Figure S5** Steady‐state affinity measurements of different VRC01 glycovariants binding FcRn.
