# Supplementary material for: Engineering the interactions between a plant‐produced HIV antibody and human Fc receptors
Source: Plant Biotechnol J. 2019 Aug 10;18(2):402–14. doi: 10.1111/pbi.13207 (PMC6953194; doi:10.1111/pbi.13207)
Supplement: Supplementary file 1 — Figure S1 Fc glycosylation profiles of the VRC01 antibody (HEK) and its plant‐made glycovariants (WT, ∆XF, Gal). Figure S2 Comparison between 1 : 1 binding interaction and ‘heterogeneous antibody with aglycosylation’ modelling for VRC01Gal antibody binding to FcγRI. Figure S3 Steady‐state affinity measurements of different VRC01 glycovariants binding FcγRIIa. Figure S4 Steady‐state affinity measurements of different VRC01 glycovariants binding FcγRIIb. Figure S5 Steady‐state affinity measurements of different VRC01 glycovariants binding FcRn. [file PBI-18-402-s001.docx]

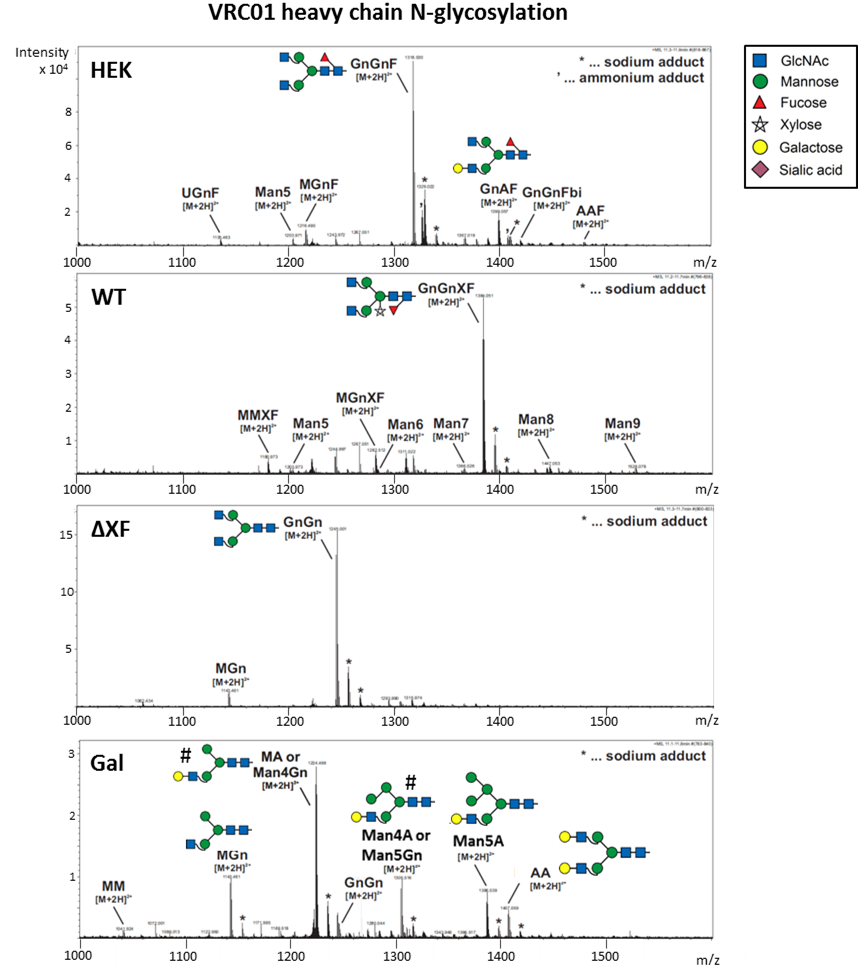


**Figure S1. Fc glycosylation profiles of the VRC01 antibody (HEK) and its plant-made glycovariants (WT, ∆XF, Gal).** Deconvoluted mass spectra of tryptic glycopeptides (EEQYNSTYR, 1189.51 Da) derived from heavy chain of different VRC01 glycovariants as determined by LC-ESI-MS. Only doubly charged ions displayed. Structures of *N*-glycans that constitute less than 5% of the total population have not been illustrated. Glycan cartoons marked with # represent the structure assumed as predominant, while abbreviations list both options. *N*-glycan abbreviations according to the ProGlycAn nomenclature.


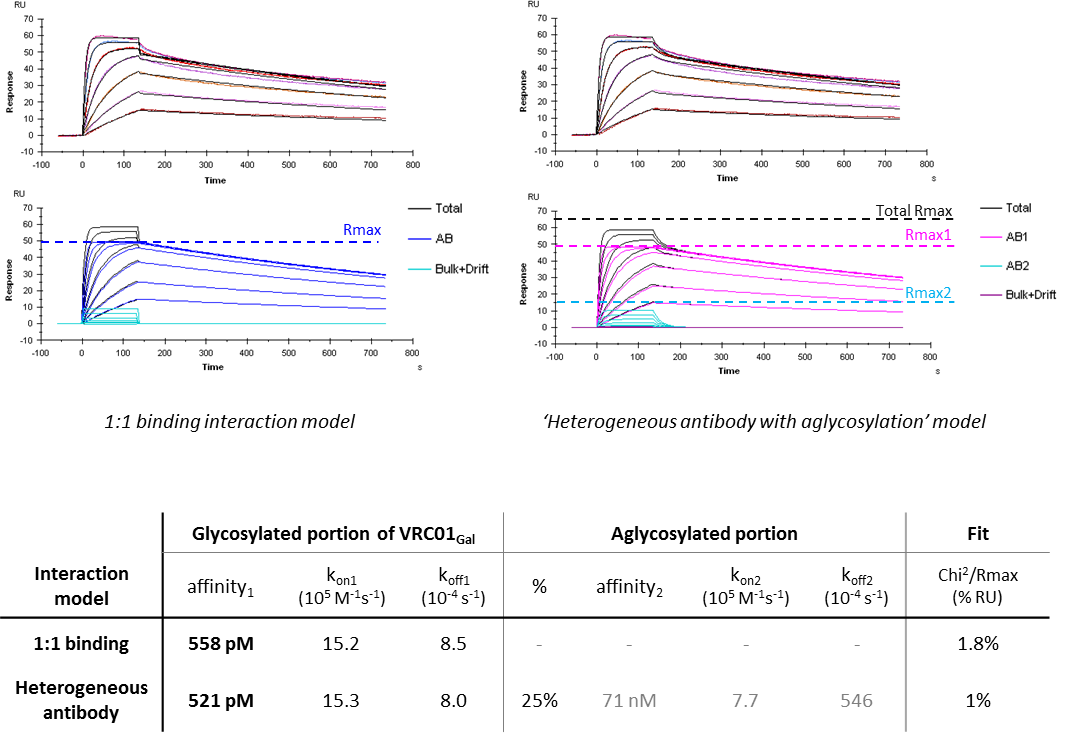


**Figure S2. Comparison between 1:1 binding interaction and “heterogeneous antibody with aglycosylation” modelling for VRC01_Gal_ antibody binding to FcγRI.** SPR sensorgrams illustrate binding and dissociation of antibody-receptor complexes. Data were analysed with either a 1:1 binding interaction model (left) or a “heterogeneous antibody with aglycosylation model (right). Dashed lines denotes maximum responses upon saturation. The results are summarised in the table. The 1:1 binding model assumes a reduced maximum response of 50 RU (dark blue lines) and any additional binding above that level (black lines) is attributed to a baseline drift (light blue lines). The ‘heterogeneous antibody with aglycosylation’ model provides a better fit to the data and any additional binding above the glycosylated portion of VRC01Gal (pink) is attributed to aglycosylated antibodies (light blue), resulting in a proper total maximum response (66 RU). Kinetics and affinity parameters for the glycosylated antibodies of the VRC01Gal are not significantly affected by the model choice.


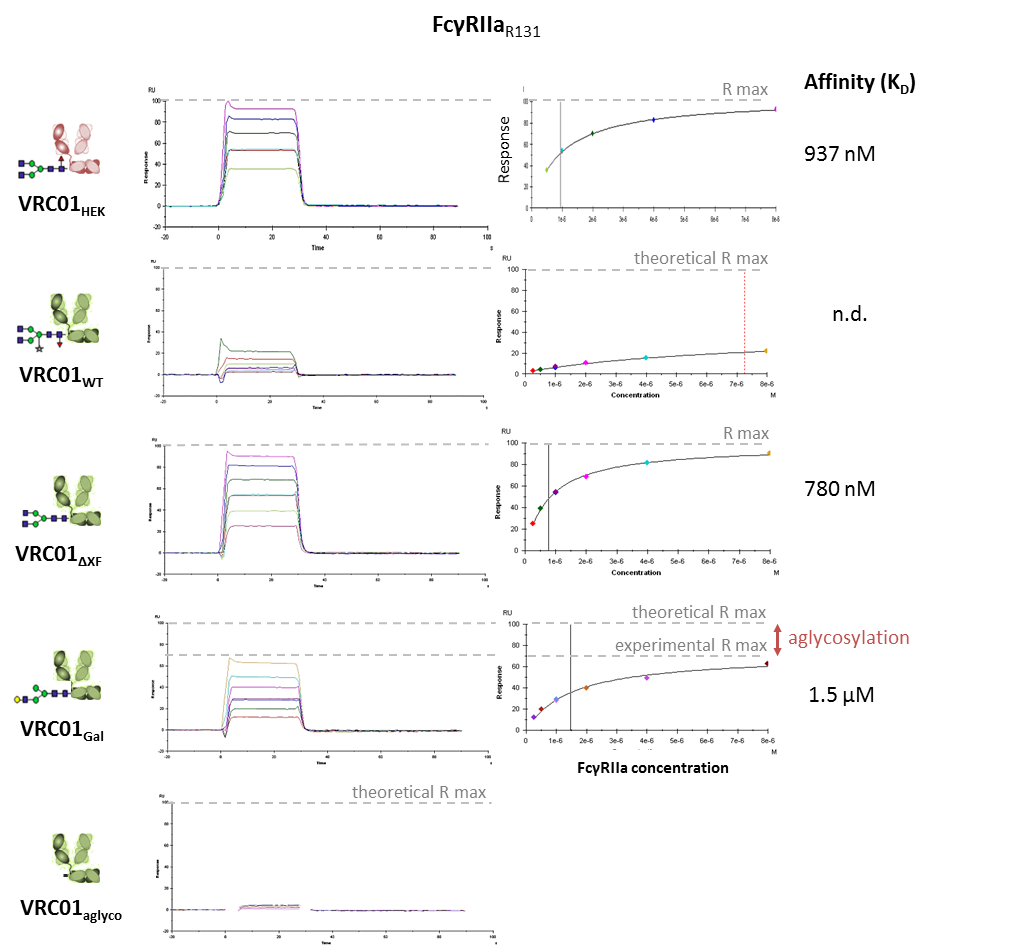


**Figure S3. Steady-state affinity measurements of different VRC01 glycovariants binding FcγRIIa.** During injection of FcγRIIa (0.5-8μM) over the Protein A captured antibodies, the analytes immediately reach a dynamic equilibrium of complex formation and dissociation, which is represented by the flat binding shown in the sensorgrams. Responses at steady-states are analysed using steady-state affinity evaluation. The Biacore Evaluation software extrapolates affinity values which are indicated by a black solid vertical line (when the estimation is robust) or a red dashed line (when the estimation is only approximate).


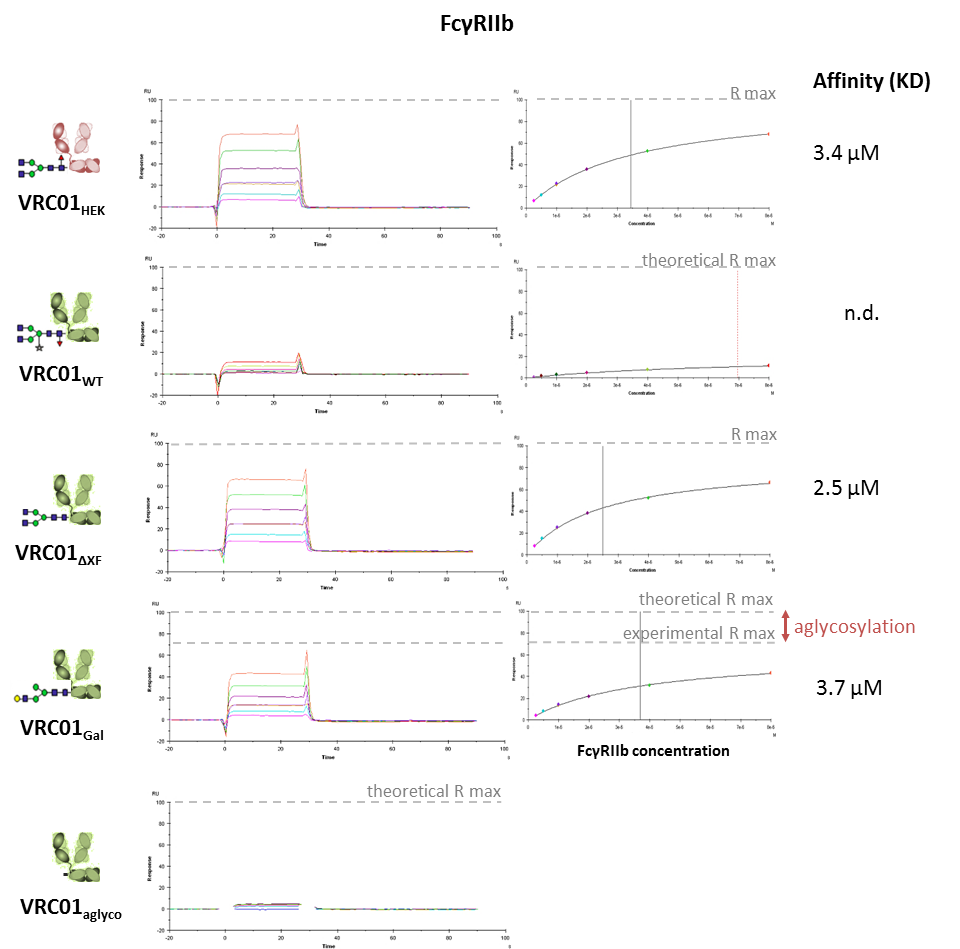


**Figure S4. Steady-state affinity measurements of different VRC01 glycovariants binding FcγRIIb.** During injection of FcγRIIa (0.5-8μM) over the Protein A captured antibodies, the analytes immediately reach a dynamic equilibrium of complex formation and dissociation, which is represented by the flat binding shown in the sensorgrams. Responses at steady-states are analysed using steady-state affinity evaluation. The Biacore Evaluation software extrapolates affinity values which are indicated by a black solid vertical line (when the estimation is robust) or a red dashed line (when the estimation is only approximate).

**
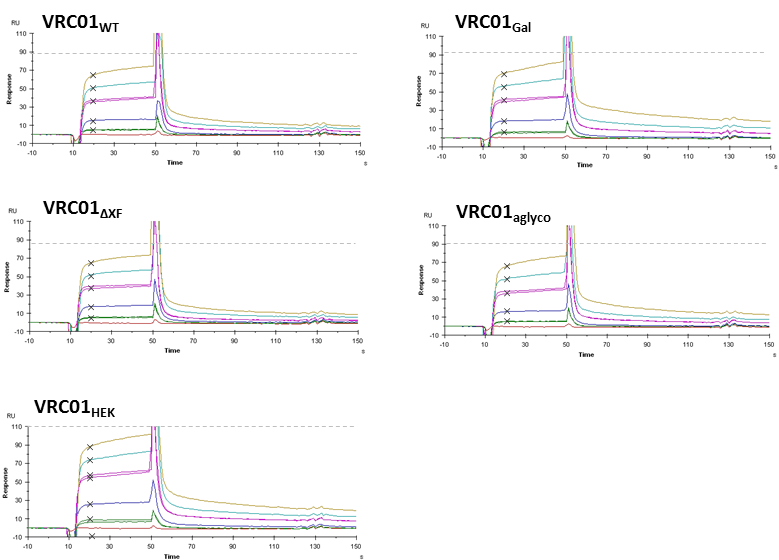
**

**Figure S5. Steady-state affinity measurements of different VRC01 glycovariants binding FcRn.**

Each VRC01 glycovariant was captured onto the anti-human Fab surface to the same level, followed by injection of recombinant human FcRn ectodomains at different concentrations (800 nM, 400 nM, 200 nM, 50 nM, 12.5 nM). Since clear steady-state equilibrium responses could not be reached, probably due to a presence of receptor dimers causing slow sensorgram upward drifts, the responses shortly after injection (as indicated by black crosses) were selected for steady-state affinity evaluation. Responses at this time point are believed to represent best 1:1 interaction between FcRn and VRC01 heavy chains, before the dimer-artefacts come into effect. Dashed horizontal lines indicate maximum response upon saturation.
